# Prospectively assessed hypothalamic-pituitary dysfunction after proton therapy in adults with head and neck, skull base and brain tumors

**DOI:** 10.1038/s41598-025-16960-z

**Published:** 2025-08-24

**Authors:** Jordan Bouter, Nathan Azemar, Anthony Vela, Pauline Dutheil, Paul Lesueur, Dinu Stefan, Yves Reznik, Juliette Thariat

**Affiliations:** 1 Department of Radiotherapy, Centre François Baclesse. 4 Av General Harris, 14000, Caen, France; 2 University Hospital of Caen, Caen, France; 3 ENSICAEN, CNRS/IN2P3, LPC Caen UMR6534, 14000 Caen, France; 4 Université de Caen Normandie, Caen, France

**Keywords:** Tumor, Brain, Head and neck, Hypopituitarism, Endocrine, Radiotherapy, Diseases of the nervous system, Oculomotor system, Endocrinology, Oncology, Pathogenesis, Signs and symptoms

## Abstract

Radiotherapy is advocated for many brain and head and neck tumors close to the pituitary gland. Pituitary hormones govern many vital functions but data of standardized monitoring for deficiencies after irradiation are lacking. We prospectively assessed the latency and frequency of hypothalamo-pituitary radiation dose-effects in patients undergoing proton therapy or mixed photon /proton beams for CNS, skull base and head and neck tumors. Radiation oncologists prospectively were asked to monitor endocrine functions based on a standardized protocol complying with international recommendations at 6, 12 months and yearly during follow-up. Patient, tumor and treatment characteristics were collected. Seventy patients (women 70%, median age 60.1 years) had undergone endocrine monitoring with a median follow-up of 20.7 (12.8–29.5) months. Thirty percent of patients had at least one pituitary deficiency before radiotherapy. Median mean dose to the pituitary gland and hypothalamus were respectively 52.0 (50.4–53.9) Gy and 24.7 (16.8–39.2) Gy. Of these patients with heavily exposed on their pituitary gland, skull base meningiomas (47.1%) and pituitary adenomas were the most represented (28.7%). Twenty-six (37.1%) patients experienced a new pituitary deficiency after a median time of 14.1 [IQR 10.3–23.6] months. Lactotroph and gonadotroph axis deficiencies occurred in 36% and 23.2% of the patients after 13.5 and 24.5 months, respectively. GH deficiency occurred in 9.7% patients after 27.1 months. TSH and ACTH axes occurred in 7.8% and 6.3% of patients after 21.8 and 19.0 months. On univariate analysis, only BMI < 25 was significantly associated with a shorter time to new deficiency onset. In this selected population of patients receiving over 50 Gy RBE to the pituitary gland, patients frequently developed at least one new pituitary deficiency within 2 years. As hormonal deficiencies are frequent after surgery and radiotherapy have functional and quality-of-life consequences and can be substituted medically, baseline and routine annual monitoring of every axis were performed. Educational programs for patients and physicians toward more systematic monitoring in patients, including those receiving < 30 Gy could serve to analyze radiation dose-effects and better predict individual endocrine normal tissue complication probabilities (NTCP).

## Introduction

Recent evidence from a meta-analysis suggest that endocrine insufficiency is very frequent in patients irradiated for brain, head and neck (HN) or skull base (SB) malignancies in the 2010–2024 period of advanced radiotherapy techniques (including intensity modulated radiotherapy) [1]. Their analysis was limited to 22 studies only, including three studies using protons, suggesting that good quality data is still limited in adults.

Patients with brain, HN/SB tumors may experience pituitary gland dysfunction by either tumor-specific or treatment-related mechanisms. Delayed detection may have significant clinical consequences as hypopituitarism has been shown to cause premature mortality and substantial morbidity [2]. Pituitary dysfunction after irradiation has been reported since the late 1980s [3]. However, dose/volume constraints to the pituitary gland have not been considered a priority in radiotherapy of brain, HN/SB tumors, perhaps because of some potential for endocrine substitution and also because of the presence of serial organs in this complex anatomic region. The pituitary is indeed a small piriform organ of 1 cm^3^ located in the sella turcica immediately under the chiasma, which is an even higher priority serial organ when planning radiotherapy because of the risk of visual loss. The distance of the tumor to these organs is critical to the HP sparing potential [4].

The recent meta-analysis showed a wide variation in hypothalamic-pituitary (HP) dysfunction, along with wide dose ranges given to the hypothalamus and pituitary, and varying follow-up times. The average prevalence for any pituitary insufficiency was 0.61; it varied significantly between the five axes and by tumor type [1]. They also observed that follow-up was not adequate and insufficiently standardized [1]. A survey was conducted among French radiation oncologists [5] to assess their practice regarding endocrine monitoring in the context of radiotherapy of adult head and neck and CNS tumors. The survey highlighted lack of adherence to monitoring and self-recognized gaps in knowledge. Systematic monitoring was recognized as a necessity for patients treated with RT for low-grade or aggressive tumors of certain tumor sites (e.g. pituitary adenomas, sinonasal tumors, nasopharyngeal tumors) only. Oncologists agreed that endocrine monitoring should be performed by an endocrinologist and should occur at least every 12 months. Agreement for monitoring specific axes of the anterior pituitary gland was stronger than for monitoring of the posterior pituitary and natremia. Finally, levels of agreement varied for replacement of deficiencies regardless of symptoms or based on symptoms for different axes of the pituitary gland. Improvements of the past 60 years have shown complex interactions and feedback loops between components of the five GH, FSH/LH, TSH, ACTH, prolactin axes or the hypothalamo-pituitary system [6]. Cellular components of the five hormonal pituitary axes are intermingled within the anterior pituitary lobe. These five axes are variably radiosensitive (i.e., their dose dependency seem to be different) and their deficiencies may occur at variable time points [7]. There are currently no guidelines specific to the radiotherapy context as to how hypothalamic-pituitary functions should be monitored and in whom based on age, tumor site, tumor proximity to the pituitary gland or hypothalamus or downstream organs or pituitary dose once radiotherapy plan has been made. More systematic data collection on correlations between dose on the pituitary gland and symptoms or endocrine dosages is warranted to define dose constraints for radiotherapy optimization and prevent radiation sequelae.

Since implementation of proton therapy in 2018 at our institution; adults with tumors near the pituitary gland have been referred to our center and treated with postoperative or definitive radiotherapy either with protons only or mixed beams of protons and photons. We prospectively examined the occurrence and latency of pituitary dose effects, including deficiencies by endocrine axis, based on their clinical data, dosimetric data and endocrine levels.

## Material and methods

### Patients

Adult patients were enrolled in a prospective cohort approved by the Institutional Review Board (French/European funding: ERDF-82 FSE2014-2020 no. 18P03532/18E01765). Informed consent was obtained from all the participants (non-opposition in compliance with MR004 regulation). The study was compliant with the GDPR regulation, and all methods were performed in accordance with the relevant guidelines and regulations.

Proton therapy was systematically advocated after multidisciplinary staff meeting and technical expert committee meeting at the Normandy proton therapy center (Caen, France). It was performed in the definitive or adjuvant setting. Patients ≥ 18 years old, with tumors (benign or malignant) of the brain or head and neck between 08/2018 and 03/2021 (initial ramp-up phase) who included provided that they underwent endocrine baseline testing and at least one follow-up testing. Past medical history, menopausal status, treatments and comorbidities were collected. In pituitary adenomas, radiotherapy was in this series performed in unresectable cases or in patients with R1/R2 resection or with multiple relapses. Exclusion criteria were age < 18, no baseline or no follow-up endocrine testing or pan-hypopituitarism before irradiation.

### Irradiation

Proton irradiations (PT) were performed using Proteus®ONE (IBA, Louvain la Neuve, Belgium). Patients who partly received photon-based radiotherapy underwent stereotactic radiotherapy (SRT) with Cyberknife® (Accuray) or intensity modulated radiotherapy (IMRT) with TomoTherapy® (Accuray) or RapidArc® (Varian) in case of PT beam downtime or as a combined treatment. Prescribed radiotherapy dose and dose-volume histograms (DVH), were collected from treatment planning systems (TPS). Tumor and organ at risk delineation were based on millimetric CT scan co-registered with contrast-enhanced fusion MRI in treatment position. Proton therapy was performed using pencil beam scanning (PBS) with a ProteusOne® machine. The first treatments were delivered in IMPT (Intensity Modulated Proton therapy) with Single field optimization (SFO) and after one year, multiple field optimization (MFO) was validated to complete the technical offering and thus offer more conformal dose distributions in the case of complex targets with greater sparing of critical organs. Robust optimization was used assuming 3 mm positioning uncertainty and 3%-range uncertainty (or higher in case of complex tissue interfaces or sinonasal cavity filling uncertainties) using the Raystation TPS (Raysearch®). Dose included a 1.1 relative biological effectiveness corrective factor. Treatment was delivered in 1.8-2 Gy (RBE) fractions, five days a week. Dose constraints to the pituitary gland were prescribed as a trade-off between tumor location and technical sparing feasibility. Combinations of protons and photons were sequential.

### Endocrine monitoring

Radiation oncologists were encouraged to systematically monitor endocrine levels at baseline before initiation of proton therapy and during follow up at 6 months, 12 months and yearly thereafter (or based on symptoms) but were left the final decision to or not to perform testing. For patients referred by an endocrinologist, endocrine follow-up was conducted by the same specialist who initiated the referral. Patients were asked to perform their blood tests at the same laboratory. To properly assess dose-effects and the impact of previous damage from tumor, surgery, or any other treatment, clinical and endocrine status were collected at baseline before PT.

Endocrine follow-up was performed in patients who had undergone pituitary surgery prior to radiotherapy in order to distinguish pre-existing deficiencies (due to the tumor or surgery) from new deficits potentially induced by radiotherapy. Baseline hormonal assessment was systematically conducted before radiotherapy to establish a reference point. Only patients without complete panhypopituitarism were included, allowing meaningful follow-up and time-to-deficiency analyses. Every anterior pituitary axis was assessed with a morning blood test: prolactin, thyroid (TSH-fT4), adrenal (ACTH-cortisol), gonadal (FSH-LH-estradiol/testosterone) and somatotropic (GH-IGF1) axis. Dynamic tests such as the insulin tolerance test or the glucagon stimulation test were performed upon discretion of the referring physician. Blood test values were normalized to laboratory ranges. Endocrine data were collected from their medical records at Centre François Baclesse or from their endocrinologist when indicated. Data interpretation relied on the Endocrine Society guidelines [8]. Hypoprolactinemia and hyperprolactinemia were defined according to laboratory thresholds. Hypogonadism was defined as low testosterone or estradiol associated with low to normal FSH and LH. In postmenopausal women, non-elevated FSH was sufficient. Hypothyroidism was identified as low fT4 associated with low to normal TSH. Cortisol deficiency was defined as plasma levels < 3–5 µg/dL and excluded for plasma levels > 150–180 µg/dl. Dynamic tests were recommended for cortisol plasma levels between 3 and 150 µg/dL. Growth hormone deficiency was identified as low IGF-1 plasma levels for age or poor response to dynamic testing.

### Statistics

Quantitative parameters were described as median and interquartile range. Qualitative parameters were expressed as frequency and percentage. Dysfunction-free survival curves and prognostic factors were estimated with the use of the Kaplan–Meier method and expressed with 95% confidence interval. The threshold for statistical significance was set to *p* < 0.05. Statistical analysis was performed using R Statistical Software (version 4.2.0; R Foundation for Statistical Computing, Vienna, Austria).

## Results

### Population characteristics

**Fig. 1:**
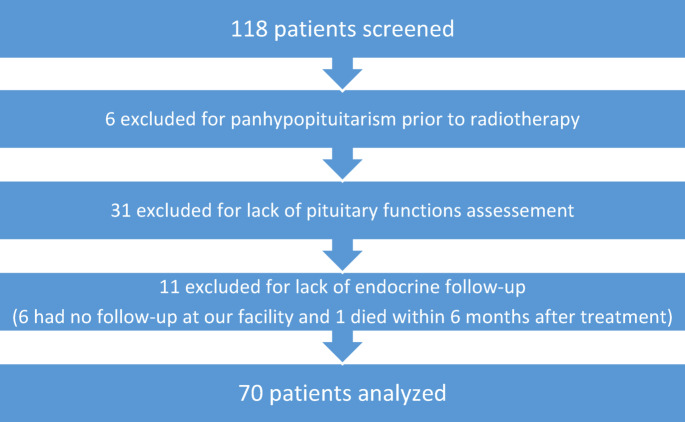
Flow chart of selected patients versus whole cohort and reasons for exclusion.

Seventy consecutive adults (59.3%) were enrolled among the 118 patients treated in the 30-month accrual period (Fig. 1). Six patients were excluded for baseline panhypopituitarism. Six patients referred by other institutions were followed by their referring physician; 30.5% of patients were excluded for lack of endocrine monitoring.

Most patients were women; median age was 60.1 years (Table 1). Most patients were WHO performance status 0–1 (94.3%) or WHO 2. Pituitary adenomas (28.7%) and meningiomas (47.1%) were the two prevalent histologies. About half (44.4%) of the pituitary adenomas were secreting. The secreted hormone was GH for 5 patients, prolactin for 1 (dual secretion of GH-prolactin) and ACTH for 3 patients. Twenty-five patients (35.7%) had definitive radiotherapy while 64.3% had undergone 1 to 3 surgical procedures before irradiation. Median time between surgery and radiotherapy was 244 (range: 142.5–453.8) days. Of note, 57.1% of the patients had visual field deficiency of any severity, indicating close proximity between the tumor and optic pathways, and therefore the pituitary gland. Also, 32.9% were already under hormone replacement therapy of at least one axis. Additionally, 35.7% and 10% were under medication for respectively high blood pressure and diabetes mellitus.

**Table 1:** Characteristics of patients, tumors, endocrine levels and antitumor treatments.

	N (%)
Sex M/F	21(30) / 49(70)
Median age	60.1 (IQR : 49.6–67) ; range : 19.7–89.6
WHO	
0	48 (68.6)
1	18 (25.7)
2	4 (5.7)
3–4	0 (0)
BMI	
< 18.5	3 (4.3)
18.5–25	27 (38.6)
> 25	37 (52.9)
NR	3 (4.3)
Histology	
Pituitary adenoma including secreting types	18 (28.7) / 8 (44.4)
Astrocytoma	1 (1.4)
Chordoma	3 (4.3)
Craniopharyngioma	2 (2.9)
Meningioma	33 (47.1)
Head and neck tumors	13 (18.6)
Visual field deficiency	
Yes	40 (57.1)
No	30 (42.9)
Hormone replacement	
Yes	23 (32.9)
No	46 (65.7)
NR	1 (1.4)
Antihypertensive drug	
Yes	25 (35.7)
No	45 (64.3)
Antidiabetic drug	
Yes	7 (10)
No	63 (90)
Number of surgeries	
0	25 (35.7)
1	25 (35.7)
2	18 (25.7)
3	2 (2.9)
Median time (days) from last surgery to end of radiotherapy	244 (IQR: 142.5–453.8)

BMI body mass index.

Sex (*p* = 0.02), prior surgery (*p* = 0.004), number of surgeries (*p* < 0.001), and hormone replacement therapy (*p* < 0.0001) varied significantly by tumor type (Table 2). Patients treated for meningioma or pituitary adenoma were predominantly women (84.8% and 61.1%, respectively). More than half of the meningioma patients received definitive radiotherapy, whereas those with pituitary adenomas more often underwent one or multiple surgeries (61.1%) and were already on hormone replacement therapy prior to radiotherapy. Notably, some patients receiving hormone replacement were treated for peripheral thyroid dysfunction, and others were on hydrocortisone following long-term corticosteroid therapy.

**Table 2:** Characteristics by tumor type.

	Pituitary adenoma	Astrocytoma	Head and neck tumors	Chordoma	Cranio-pharyngioma	Meningioma	p
Male / Female	7 (38.9)/ 11 (61.1)	0 / 1 (100)	5 (38.5)/ 8 (61.5)	2 (66.7)/ 1 (33.3)	2 (100) / 0	5 (15.2)/ 28 (84.8)	0.0235
Age (years)	61.4	32.6	48.3	73.5	44.5	60.9	
WHO							0.2795
0	15	1	7	2	1	22	
1	3	0	4	1	0	10	
2	0	0	2	0	1	1	
BMI							0.4814
< 18.5	1 (5.9)	0	2 (15.4)	0	0	0	
18.5–25	6 (35.3)	1 (100)	5 (38.5)	1 (33.3)	0	14 (45.2)	
> 25	10 (58.8)	0	6 (46.2)	2 (66.7)	2 (100)	17 (54.8)	
Number of surgical procedures	0.0006						
0	2 (11.1)	0	4 (30.8)	0	0	19 (57.6)	0.0047
1	5 (27.8)	1 (100)	8 (61.5)	2 (66.7)	0	9 (27.3)	
2	9 (50.0)	0	1 (7.7)	1 (33.3)	2 (100)	5 (15.2)	
3	2 (11.1)	0	0	0	0	0	
Hormone replacement	0.0007						
≥ 1	12 (66.7)	0	1 (8.3)	0	2 (100)	8 (24.2)	
0	6 (33.3)	1 (100)	11 (91.7)	3 (100)	0	25 (75.8)	

Pituitary dysfunction was either not reported or not interpretable for 10% to 23% of the hormonal axes. For example, in patients with pituitary adenoma (N = 4) receiving cabergoline, prolactin function cannot be interpreted. Prior to radiotherapy, pituitary deficiency in at least one hormonal axis was identified in 20 patients (28.6%). Prolactin secretion was altered in 13 patients (18.6%), TSH in 5 (7.1%), ACTH in 3 (4.3%), FSH/LH in 7 (10%), and GH in none (Table 3).

**Table 3:** Pituitary dysfunction at baseline.

	N (%)
Any axis	
Yes	20 (28.6)
No	50 (71.4)
Lactotropic	
Yes	13 (18.6)
No	45 (64.3)
Not reported or not interpretable	12 (17.1)
Thyreotropic	
Yes	5 (7.1)
No	58 (82.9)
Not reported or not interpretable	7 (10)
Corticotropic	
Yes	3 (4.3)
No	60 (85.7)
Not reported or not interpretable	7 (10)
Gonadotropic	
Yes	7 (10)
No	52 (74.3)
Not reported or not interpretable	11 (15.7)
Somatotropic	
Yes	0
No	54 (77.1)
Not reported or not interpretable	16 (22.9)

### Treatment parameters

Standard doses were prescribed based on tumor histology. For example, the prescribed dose was 50–54 Gy for pituitary adenomas, 54–59.5 Gy for intermediate-to-aggressive meningiomas, 52.2–54 Gy for craniopharyngiomas, and 60–70 Gy for HN tumors. Among the patients with HN tumors, 5 had nasopharyngeal carcinoma, 4 had sinonasal tumors, 2 had oropharyngeal carcinoma, and 2 had other locations (including parapharyngeal and laryngeal primaries). Notably, the nasopharyngeal and sinonasal cases, which are anatomically closer to the hypothalamo-pituitary axis, received higher doses to the pituitary and hypothalamus. Twenty-two patients (31%) received a combination of protons and photons, while the remaining 69% received protons only. Dosimetric parameters for three organs at risk—the pituitary gland, hypothalamus, and optic chiasm—as well as for the clinical target volume (CTV), are summarized in Table 4. The pituitary gland received a median mean dose of 52 Gy (range: 0.1–77.4 Gy) and D2% of 53.5 Gy (range: 0.3–83.3 Gy). This was approximately 2 Gy higher than the dose to the optic chiasm and about 1.5 Gy lower than the dose to the CTV, reflecting the close proximity between the target volume and the pituitary gland. In contrast, the hypothalamus received a median mean dose of 24.7 Gy (range: 0–50 Gy) and D2% of 43.0 Gy (range: 0–59.4 Gy). In cases of pituitary adenomas, the pituitary gland could not be delineated and its dose was therefore assumed to be equal to that of the CTV, given the homogenous dose distribution characteristic of proton therapy.

**Table 4:** Treatment dose characteristics.

Dose	D95% (IQR)	D50 (IQR)	DMean (IQR)	D2% (IQR)	NR
Pituitary gland	50.2 (38.2–52.4) Range : 0.0–67.5	52.1 (47.3–53.4) Range : 0.1–77.7	52.0 (50.4–53.9) Range : 0.1–77.4	53.5 (51.3–56.9) Range : 0.3–83.3	0
Hypothalamus	6.0 (1.8–18.9) Range : 0.0–45.2	22.9 (9.8–40.7) Range : 0.0–55.5	24.7 (16.8–39.2) Range : 0.0–50.0	43.0 (32.2–50.9) Range : 0.0–59.4	8
Optic chiasma	47.6 (33.0–50.1) Range : 0.0–52.3	49.9 (44.1–51.3) Range : 0.0–53.8	49.6 (43.1–50.8) Range : 0.0–53.2	50.9 (49.3–52.0) Range : 0.0–54.6	3
CTV	51.8 (49.8–52.6) Range : 49.0–64.1	53.8 (50.7–53.9) Range : 50.4–69.6	53.6 (50.6–53.9) Range : 50.4–68.8	55.2 (52.3–55.7) Range : 51.5–72.2	0

## Outcomes

### Clinical outcomes

The median follow-up was 20.7 months (IQR: 12.8–29.5), with a median of 2.0 endocrine assessments per patient (IQR: 1–3). The vast majority of patients demonstrated stable disease or partial response on their most recent MRI. Tumor progression occurred in two patients (2.9%) one year after completing radiotherapy; their histologies were somatotropic pituitary adenoma and craniopharyngioma. An additional patient (1.4%) with meningioma experienced tumor progression two years post-treatment. Notably, one patient with a corticotropic pituitary adenoma became pregnant within a year of radiotherapy, and the pregnancy was successful.

Five patients died during follow-up, with a median age at death of 73 years (range: 62–91). Of these, three had no diagnosis of pituitary deficiency. Three patients were treated for meningioma, while the other two had aggressive pituitary tumor and chondrosarcoma, respectively. Only the latter two showed evidence of tumor progression. The patient with the aggressive pituitary tumor developed panhypopituitarism, including hypoprolactinemia, within six months of completing radiotherapy.

### Endocrine dysfunctions

Twenty-six patients (37.1%) developed a new pituitary dysfunction in at least one hormonal axis after a median of 14.1 months (IQR: 10.3–23.6) (Table 5). Among the 20 patients (28.6%) with at least one hormonal axis deficiency prior to radiotherapy, 12 (60%) developed additional deficiencies during follow-up. These included new deficits in the gonadotropic (n = 5), lactotropic (n = 4), thyreotropic (n = 2), and somatotropic (n = 1) axes. Notably, no patient in this subgroup experienced normalization of previously impaired axes. Among the 35 patients tested within the first 6 months, 6 (17.1%) developed a new deficiency. The lactotroph and gonadotroph axes were the most frequently affected, with dysfunction occurring in 35% (hyperprolactinemia) and 23.2% of patients, respectively, after a median of 13.5 months (IQR: 8.4–22.4) and 24.5 months (IQR: 15.9–30.0).

**Table 5:** Incidence of new pituitary dysfunctions during follow-up.

	Incidence (% patients)					Median time to deficiency (months) [IQR]
	Any time point	0–6 months	6–12 months	1–2 years	2–3 years	
Any axis	26/70 (37.1%)	6/35 (17.1%)	9/42 (21.4%)	10/28 (35.7%)	1/12 (8.3%)	14.1 [IQR 10.3–23.6]
Prolactine	17/49 (35%)	4/26(15.4%)	7/28(25%)	7/16 (43.8%)	0/3	13.5 [8.4–22.4]
TSH	5/64 (7.8%)	0/29	2/39 (5.1%)	1/24 (4.2%)	2/9 (22.2%)	21.8 [12.9–34.4]
ACTH	2/62 (3.2%)	0/27	0/35	1/24 (4.2%)	1/10 (10%)	270 [24.0–30.0]
FSH/LH	13/56 (23.2%)	2/25(8%)	2/31 (6.5%)	6/23 (26.1%)	3/8 (37.5%)	24.5 [15.9–30.0]
GH	6/62 (9.7%)	1/28 (3.6%)	0/36	2/23 (8.7%)	3/11 (27.3%)	27.1 [22.5–35.4]
Compliance		50%	60.9%	70%	85.7%	

Two patients (2.9%) developed hypoprolactinemia. One was treated for a spindle cell oncocytoma, developed panhypopituitarism within 6 months, and died one year after irradiation. The second received proton therapy for a meningioma and had deficiencies in three hormonal axes at the last follow-up. All 13 patients with FSH/LH deficiency were women, and 11 of them also had hyperprolactinemia. GH deficiency occurred in 9.7% of patients after a median of 27.1 months (IQR: 22.5–35.4). The TSH and ACTH axes were the least frequently impaired, affecting 7.8% and 3.2% of patients, respectively, after a median of 21.8 months (IQR: 12.9–34.4) and 27.0 months (IQR: 24.0–30.0). However, the first cases of TSH and ACTH deficiencies appeared within the first year post-treatment.

Most dysfunctions were diagnosed between 6 months and 2 years after the end of proton therapy. No spontaneous normalization of any hormonal axis was observed during follow-up. Compliance with scheduled endocrine assessments improved over time, rising from 50% at 6 months to 85.7% at 3 years post-irradiation.

We further examined whether the type of radiotherapy, protons only (n = 48, 69%) versus combined protons and photons (n = 22, 31%), influenced the occurrence of new pituitary deficiencies. New hormonal deficits occurred in 18/49 (36.7%) patients treated with protons only, and in 8/22 (36.4%) patients treated with combined modalities. No significant difference in time to deficiency or specific axis involvement was observed between groups.

**Fig. 2:**
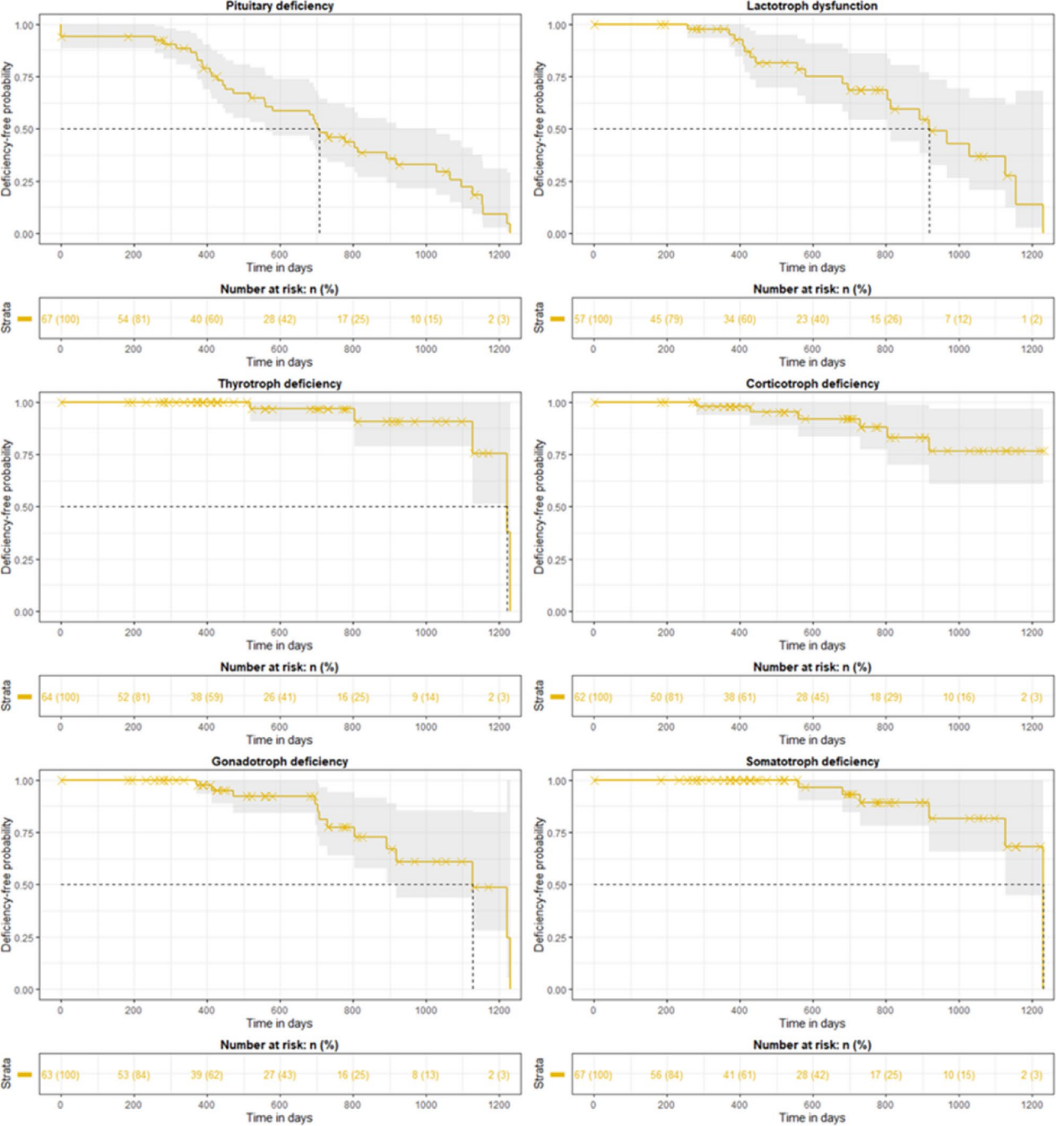
Kaplan–Meier representation of deficiency-free probability for each axis.

Dysfunction-free probabilities are illustrated in Fig. 2. At the median follow-up, approximately 40% of patients remained evaluable for each hormonal axis. The risk of developing a new pituitary deficiency reached 50% at 23 months.

Among the prognostic factors, only a BMI < 25 was significantly associated with a shorter time to onset of new pituitary deficiency (Table 6). Tumor type, except for PG on univariate analysis, did not appear as a prognostic factor but some tumors were rare in the cohort. Among the 5 NPC patients in our cohort, 3 developed at least one new pituitary deficiency during follow-up.

**Table 6:**
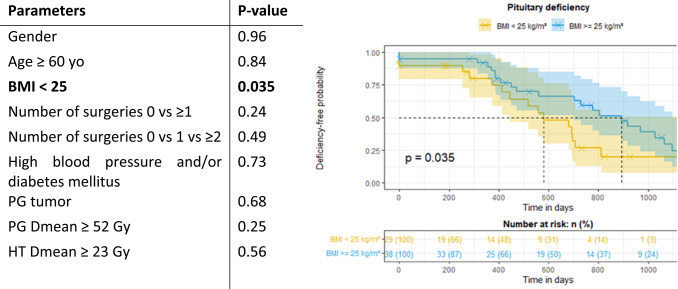
Prognostic factors of at least one anterior pituitary deficiency. Kaplan-Meier curve of deficiency-free probability for patients with BMI below or above 25 kg/m²

**Fig. 3:**
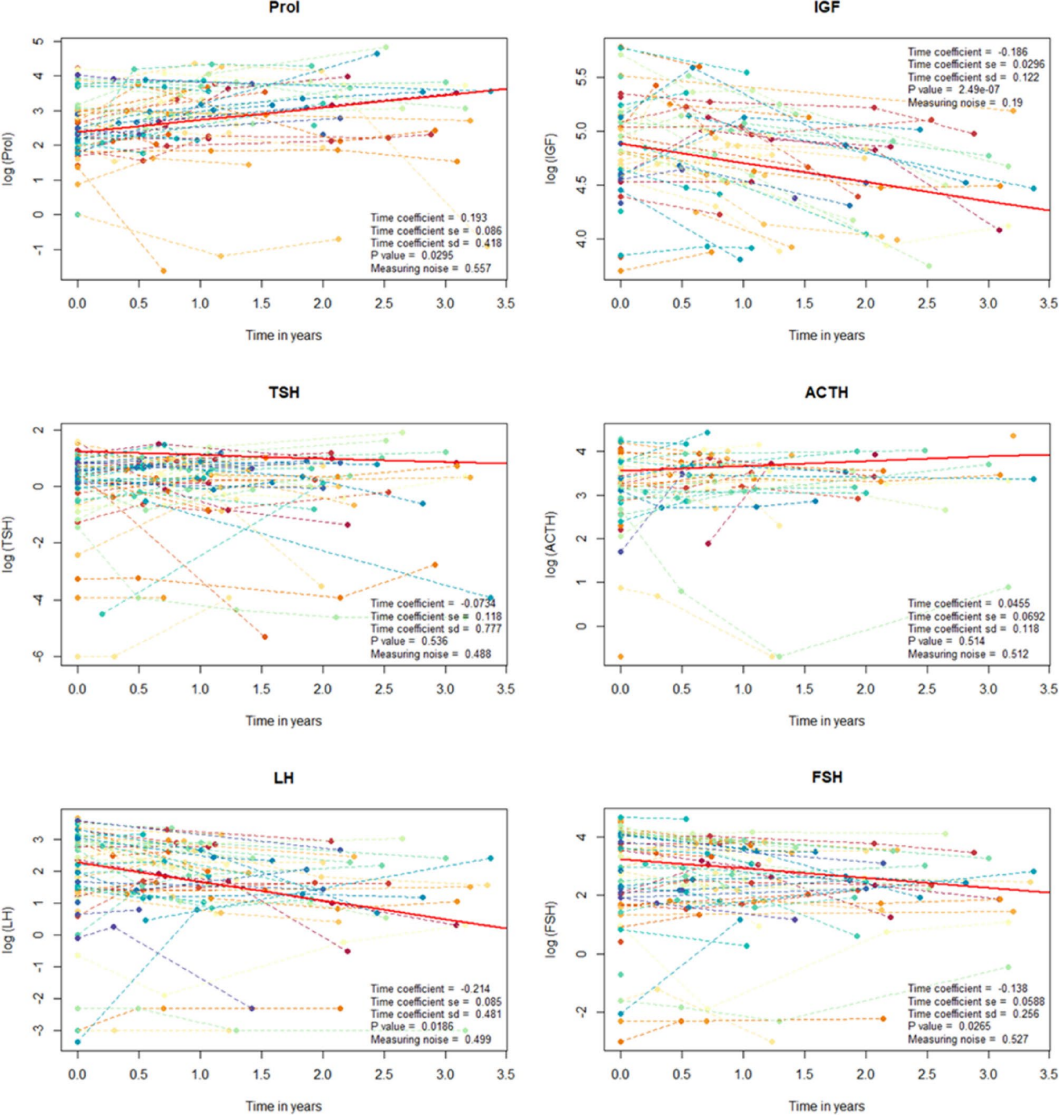
Evolution of plasma levels for each pituitary hormone. Each color represents an individual patient, while the red line indicates the trend estimated by the mixed linear model. Patients were censored after their last endocrine assessment. Number at risk is the number and percentage of patients still evaluable at a given time.

Using raw plasma levels (Fig. 3), we applied a mixed linear model to evaluate overall changes in hormone levels across the cohort. Prolactin levels significantly increased over time following irradiation (*p* = 0.0295), while IGF, FSH, and LH levels significantly decreased (*p* = 2.49 × 10⁻⁷, *p* = 0.0186, and *p* = 0.0265, respectively). The mixed linear model did not show a significant change over time for TSH or ACTH levels.

## Discussion

Our study reported a high rate of patients developing new pituitary deficiencies, up to 37.1% of 70 patients with a median follow-up of 20.7 months. These deficiencies could occur within the first six months following the end of radiotherapy. Few prospective studies have focused on pituitary deficiencies after irradiation and all of them used photons. Despite a short follow-up compared to retrospective studies, our cohort is one of the largest among prospective studies and the only one including multiple histologies. Similar to other studies, we report a high frequency of HP deficiencies [1, 9].

Median age was 60 years in our cohort as opposed to 47 years on average in other studies. Women were more frequent than men, which was related to predominance of meningiomas in this series. Patients irradiated for nasopharyngeal carcinoma (NPC) had the highest risk with a mean prevalence of 0.68 [1]. Dose constraints to the pituitary gland and hypothalamus were left to physician’s appreciation. In monitored patients, most pituitary glands received a mean dose > 50 Gy which prevented identification of deterministic effects and thresholds. Indeed, a mean PG dose > 50 Gy has been identified as a risk factor for pituitary deficiency in multivariate analysis [10]. Similarly, a correlation between the dose to the pituitary and the occurrence of hormonal axis insufficiency could not be observed [1], possibly because testing was primarily conducted in at-risk patients. In this population of patients receiving > 50 Gy to their PG per selection on endocrine monitoring, mean HT dose was not a risk factor (*p* = 0.56) although Partoune et al.reported a risk progressively increasing without a clear threshold [11]. It has been described that the higher the dose to the pituitary, the shorter the latency of GH deficiency [7]. This effect has never been reported for the other axes. However, it could be envisaged that it exists to a lesser extent due to a lower radiosensitivity. Larger prospective studies with a wider range of doses and regular endocrine assessments would be necessary to clarify its importance. Kyriakakis et al*.* described dose thresholds for every axis, from 10 Gy for GH to 50 Gy for TSH [12] and Darzy et al*.* reported mostly isolated GH deficiencies below 30 Gy [7].

Our data of all five axes show that 37.1% of patients developed any new pituitary dysfunction within a median time of 14.1 months, including 17.1% within the first 6 months. Endocrine deficiencies have been reported as early as 3 months after radiotherapy, even at doses below 30 Gy [13]. We showed that pituitary deficiency occurred in at least one hormonal axis in 28.6% of cases and the number of surgical procedures before radiotherapy was an important risk factor; suggesting that it is critical to assess follow up levels with respect to baseline levels. Interestingly, among the 20 patients with deficits before radiotherapy, there was a trend toward cumulative or progressive pituitary dysfunction in patients with pre-existing impairment, consistent with a possible additive effect of surgery and irradiation. Moreover, repeated surgical interventions may increase the risk of damage to the pituitary gland or stalk, compromise vascular supply, or lead to scarring and adhesions that exacerbate radiation effects. This cumulative burden may explain the higher frequency of new deficits observed in patients with multiple surgeries [14]. Finally, GH dysfunction was 9.7% within 27.1 months, lactotroph dysfunction 35% within 13.5 months, gonadotroph 23.2% within 24.5 months, ACTH 3.2% with 27.0 months and TSH 7.8% within 21.8 months. In a recent meta-analysis, mean prevalence of endocrine insufficiency of 1 axis was 19%, 2 axes 22%, 3 axes 5% and panhypopituitarism 17% [1], of overall similar magnitude as in our cohort. Mean prevalence of GH deficiency was 40%, of higher magnitude than in our series, prolactin 22% of slightly lower magnitude, gonadotropin 20% of similar magnitude, while ACTH 16% and TSH 16% of higher magnitude than in our series [1]. A significant correlation is indeed observed between any endocrine insufficiency and follow-up time [1] and could explain the slight differences. Pituitary axes show different radiosensitivity in our series and the literature [1] and long term data are warranted for more personalized replacement therapies [7, 14, 15] and to promote long-term follow-up as HP deficiencies frequently occur after some years [10]. Identified differences in sensitivity vary between the anterior pituitary axes, with the growth hormone axis being the most easily damaged by irradiation and the thyroid hormone axis the least sensitive. Pituitary gland protection and early detection of deficiencies need further investigations. Of note, the hypothalamus seemed to be more vulnerable to radiation dose compared to the pituitary gland, which warrants further systematic, standardized and long-term monitoring data to establish reliable normal tissue complication probability (NTCP) models for HP deficiencies [1].

The rationale for using protons over photons lies in their particular dose deposition. The superiority of proton therapy lies in their excellent distal dose fall off at the end of the Bragg peak with virtually no dose behind, and this is particularly interesting to spare substantial volumes of distant organs. However, for relatively superficial tumors on the CNS and HN, most commercial pencil beam scanning proton therapy machines use a minimal 100 MeV, which requires the use of a range shifter to reach tumors less than 7.5 cm below the skin. Such energy degraders widen the lateral penumbra [16]. In tumors abutting the pituitary gland or the optic chiasm, stereotactic irradiation was sometimes used as the boost component to yield the steepest gradient-generating technique. Proton therapy however significantly spares more distant organs and the brain itself, which can have significant advantages on cognition compared to photons. Hypothalamic-pituitary deficiencies after proton therapy were described in two papers [17, 18]. In these retrospective studies of 74 (Lamba *et* al.) and 103 patients (De Marzi et al.), patients were treated for meningioma, chordoma or chondrosarcoma with a median mean dose to pituitary gland of 51.4 Gy and 54 Gy. Lamba et al. reported a new deficiency rate of 20% for any axis, occurring at a median time of 0.9 to 2.7 years after radiation depending on the axis. No difference was found between protons and photons regarding pituitary deficiencies. De Marzi et al. reported a 44% rate for any axis but no information regarding the duration of the follow-up was available. We conducted a comprehensive analysis of endocrine deficiencies and found that new deficits occurred in 40% of patients undergoing radiotherapy within a median follow-up of 5.6 years [19], in line with a more recent study [1] and the time-effect relationship. We observed similar rates of pituitary dysfunction between patients treated with protons alone and those who received combined photon-proton therapy. This may reflect comparable radiation doses delivered to the hypothalamo-pituitary axis across modalities, particularly in anatomically complex cases where sparing was limited regardless of technique. Few studies reported hypothalamic-pituitary deficiencies after proton therapy but no difference was found between proton-based and photon-based irradiations of tumors close to the PG, suggesting similarly steep gradient gradients.

A limitation of our study could be the absence of systematic dynamic tests, which probably led to a significant underestimation of the rate of somatotrophic and corticotropic partial deficiencies. However, a recent study of 246 patients referred to endocrinologists after cranial radiotherapy showed ACTH deficit was rare, and never isolated. The authors suggested that it may not be necessary to carry out a dynamic test for ACTH if no other deficits are diagnosed [15]. Another issue is GH supplementation in adults, especially in the context of a tumor. It remains widely debated, which might be one of the reasons why these deficiencies are not optimally investigated in routine practice [20]. GH replacement seemed to improve well-being parameters in adults but there were safety concerns [20, 21] about whether GH replacement increases future cancer risk. It is difficult to identify factors that modulate cancer risk in adults and there is some evidence for in-vitro pro-neoplastic properties and increased serum concentrations of IGF-I that could independently contribute to worse tumor or mortality outcomes in at-risk populations. Similarly, radiation-induced gonadotropic deficits may be overestimated in the absence of correction of associated asymptomatic hyperprolactinemia. We found that BMI < 25 kg/m^2^ is associated with earlier pituitary deficiency. To our knowledge, this association has never been reported and currently lacks pathophysiological explanation. The interpretation of prolactinemia may be particularly complex in this oncology context. This can only emphasize the need for more systematic and long-term assessment of the HP axes in oncology adults [22]. However, we showed a relative lack of patient adherence to follow-up of long-term side effects, particularly if asymptomatic. A limitation of our study and many others lies in a selection bias toward monitoring endocrine deficiencies only in patients at high risk of deficiency. Some patients were lost to follow-up at our facility or did not want to continue endocrine monitoring. At 3 years, 14 patients were evaluable and achieved over 85% compliance. This is common in oncology and might be improved with better education of patients, radiation oncologists and family doctors to promote a long-term standardized follow-up of those patients, considering that blood testing is hardly invasive. To overcome sampling and quantification biases, we considered using the raw data of plasma hormone levels from the cohort to describe the effects of irradiation. This approach provides additional information and has never been described in the context of hypothalamic-pituitary deficiencies. Moreover, it could be a first step towards modeling radiation-induced hypothalamic-pituitary effects. We included patients with pituitary resection and pre-existing pituitary deficits in the analysis, as they are at high risk of further deterioration, as long as they did not have complete panhypopituitarism. Monitoring this subgroup provides valuable insight into the potential additive or progressive impact of radiotherapy on residual pituitary function, which reflects real-world clinical scenarios and underscores the importance of individualized long-term endocrine follow-up.

The fact that this series is performed in patients undergoing proton therapy should not be misunderstood as an investigation of proton-specific side effects. More than ever, comparative studies and randomized photons/protons trials are warranted. Additionally, endocrine management guidelines [8] customized to the adult oncology context would be helpful to better standardize endocrine monitoring, its duration, based on the tumor characteristics and dose level, and replacement therapies in routine care [11, 12].

## Conclusion

Hypothalamic-pituitary deficiencies are frequent and vary by axes. Guidelines for monitoring of endocrine deficits during and after radiotherapy are warranted to ensure that patients receive the necessary endocrine substitution when needed to maintain their quality of life and critical functions. Owing to their functional and quality-of-life consequences, and possible hormonal replacement, patient and physician education is warranted so that baseline and endocrine monitoring of every axis be performed after multimodal treatments. More systematic monitoring in patients receiving lower doses could also serve to better analyze radiation dose-effects and predict individual endocrine normal tissue complication probabilities (NTCP).

## Data Availability

The datasets generated and/or analyzed during the current study are not publicly available due to French regulation but are available from the corresponding author on reasonable request.
